# *Scutellaria barbata* D. Don extract inhibits the tumor growth through down-regulating of Treg cells and manipulating Th1/Th17 immune response in hepatoma H22-bearing mice

**DOI:** 10.1186/s12906-016-1551-9

**Published:** 2017-01-13

**Authors:** Xuefeng Kan, Wanli Zhang, Ruxu You, Yanfeng Niu, Jianrong Guo, Jun Xue

**Affiliations:** 1 Department of Radiology, Union Hospital, Tongji Medical College, Huazhong University of Science and Technology, 1277 Jiefang Road, Wuhan, 430022 China; 2 Cancer Center, Union Hospital, Tongji Medical College, Huazhong University of Science and Technology, 1277 Jiefang Road, Wuhan, 430022 China; 3 Department of Pharmacy, Union Hospital, Tongji Medical College, Huazhong University of Science and Technology, 1277 Jiefang Road, Wuhan, 430022 China; 4 Department of Gastrointestinal Surgery, Union Hospital, Tongji Medical College, Huazhong University of Science and Technology, Wuhan, 430022 China

**Keywords:** *Scutellaria barbata* D. Don extract (SBE), Hepatoma, Immunomodulatory, H22, IL-17A, Treg cells, Th1/Th17

## Abstract

**Background:**

Previous studies showed *Scutellaria barbata* D. Don extract (SBE) is a potent inhibitor in hepatoma and could improve immune function of hepatoma H22-bearing mice. However, the immunomodulatory function of SBE on the tumor growth of hepatoma remains unclear. This study aimed to investigate the anti-tumor effects of SBE on hepatoma H22-bearing mice and explore the underlying immunomodulatory function.

**Methods:**

The hepatoma H22-bearing mice were treated by SBE for 30 days. The effect of SBE on the proliferation of HepG2 cells in vitro, the growth of transplanted tumor, the cytotoxicity of natural killer (NK) cells in spleen, the amount of CD4^+^CD25^+^Foxp3^+^ Treg cells and Th17 cells in tumor tissue, and the levels of IL-10, TGF-β, IL-17A, IL-2, and IFN-γ in serum of the hepatoma H22-bearing mice was observered. IL-17A was injected to the SBE treated mice from day 9 post H22 inoculation to examine its effect on tumor growth.

**Results:**

SBE treatment inhibited the proliferation of HepG2 cells in vitro with a dose-dependent manner and significantly suppressed the tumor growth of hepatoma H22-bearing mice. Meanwhile, it increased NK cells’ cytotoxicity in spleen, down-regulated the amount of CD4^+^CD25^+^Foxp3^+^ Treg cells and Th17 cells in tumor tissue, and decreased IL-10, TGF-β, and IL-17A levels (*P* < 0.01) whereas increased IL-2 and IFN-γ levels (*P* < 0.01) in the serum of hepatoma H22-bearing mice. Moreover, administration of recombinant mouse IL-17A reversed the anti-tumor effects of SBE.

**Conclusion:**

SBE could inhibit the proliferation of HepG2 cells in vitro. Meanwhile, SBE also could inhibit the growth of H22 implanted tumor in hepatoma H22-bearing mice, and this function might be associated with immunomodulatory activity through down-regulating of Treg cells and manipulating Th1/Th17 immune response.

## Background

Hepatocellular carcinoma (HCC) is one of the most common human cancer that shows relatively poor prognosis and rapid progression [1, 2]. The selection of HCC treatment depends on the tumor biological behavior, heterogeneity and liver function [3]. Faced with palliative care, Chemotherapy is one of the main methods. However, it may cause severe side-effects and often lead to multidrug resistance [4]. Therefore, many cancer patients tried to use Chinese herbal therapies, and several herbs have been found to have antitumor activity and become the main sources of anti-cancer drugs [5].

x*Scutellaria barbata* D. Don (SB), is a perennial herb which is natively distributed in northeast Asia. This herb was known in traditional Chinese medicine as Ban-Zhi-Lian and has been used as an anti-inflammatory and anti-tumor agent [6, 7]. It was reported that flavonoids and scutebarbatines are the main components of SB [8–10]. *Scutellaria barbata* D. Don extract (SBE) has been shown to have inhibitory effects on numerous human cancers, including hepatoma, lung cancer, colon cancer, skin cancer [11–14]. The results of previous study showed that SBE could inhibit the growth of hepatoma H22 cells in vitro and in vivo, and improve immune function of the H22 tumor bearing mice [15]. However, the immunomodulatory function of SBE on the tumor growth of HCC remains unclear and needs to be further investigated.

Previous studies have shown that tumor cells can recruit regulatory cells (Treg) to inhibit antitumor immunity in the tumor microenvironment, thus limiting the efficiency of cancer immunotherapy [16, 17]. The role of IL-17 and the IL-17 producing Th17 cells in cancer has recently become the focus of extensive investigation [18, 19]. The proportion of Th17 cells was significantly higher in HCC [20]. In the present study, we sought to examine the effects of SBE on innate immunological cells, regulatory T cells and Th17 cells in hepatoma H22-bearing mice. Our data indicated that SBE could inhibit tumor growth of hepatoma H22-bearing mice through modulating the immune function. Our findings further provided experimental evidence for the application in the treatment of HCC.

## Methods

### Overall study design

Firstly, the proliferation of HepG2 cells after SBE treatment was assessed by 3-(4,5-dimethylthiazol-2-yl)-2,5-diphenyltetrazolium bromide (MTT) assay. Secondly, 40 hepatoma H22-bearing mice were randomly divided into 4 groups (*n* = 10) and administered for 30 days: vehicle control group (0.9% saline solution); SBE treatment (50, 100, and 150 mg/kg/day). The growth of transplanted tumor in the 4 groups were observered by 3D high frequency color ultrasound (GE Healthcare, Milwaukee, WI, USA) every other day. Thirty days later, the mice were sacrificed. The concentrations of TGF-β, IL-10, IL-2, IFN-γ and IL-17A in the serum of mice were measured by ELISA. Meanwhile, the cytotoxicity of natural killer (NK) cells in spleen and the amount of CD4 + CD25 + Foxp3+ Treg cells and Th17 cells in tumor tissue were observered. Thirdly, 30 hepatoma H22-bearing mice were randomly divided into 3 groups (*n* = 10) and administered for 30 days: vehicle control group (0.9% saline solution); SBE treatment group (150 mg/kg/day); SBE (150 mg/kg/day) combined with IL-17A (0.5 ug/mouse) treatment group. The growth of transplanted tumor in the 3 groups were observered by 3D high frequency color ultrasound every other day.

### Preparation of SBE

The dried rhizomes of SB were purchased from Nanjing Haiyuan Chinese medicine decoction pieces Co., Ltd. (Nanjing, China). And they were identified by Ruxu You, from the department of pharmacology, Union Hospital, Tongji Medical College, Huazhong University of Science and Technology. The specimens were deposited in our laboratory under standard conditions. Briefly, the dried rhizomes of SB were ground into powder. The powder (1 kg) was extracted with double-distilled water (1000 mL) by reflux extraction for 1.5 h/time 2 times. Ninety-five percent ethanol (v/v) added to the combined extract to adjust the final concentration of ethanol to 85% (v/v). The precipitated polysaccharide component was removed by the fltration device. The remaining solution was concentrated at 50 °C in a rotary evaporator under reduced pressure. Finally, the extract was redissolved in methanol for high performance liquid chromatography (HPLC) analysis.

### HPLC analysis

SBE was analyzed on a Pump-L 2130 HPLC system (Hitachi, Tokyo, Japan) using an Agilent TC-C18 column (4.6 mm × 150 mm, 5 μm). The mobile phase gradient conditions consisted of methanol (A) and water (B): 0–2min, 10–10%A; 2–20min, 10–20%A; 20–40min, 20–20%A; 40–60min, 20–30%A; 60–70min, 30–60%A; 70–114min, 60–114%A. The low rate was 0.8 mL/min and the column temperature was maintained at 25 °C. Absorbance was measured at 264 nm.

### Cell culture

Human hepatocellular carcinoma cell line (HepG2) and mouse hepatocellular carcinoma cells line (H22) were obtained from ATCC (American type culture collection). Cells were cultured in dulbecco’s modified eagle media (Invitrogen, Carlsbad, CA, USA) supplemented with 10% fetal bovine serum. Human erythroleukemia cell line K562 cells were maintained in RPMI 1640 medium supplemented with 10% FBS. All cells were maintained at 37 °C in a humidified incubator gassed with 5% CO_2_.

### Reagents

Carboxyfluorescein succinimidyl ester, propidium iodide, PMA, ionomycin, monensin and collagenase were purchased from Sigma Company (Sigma, St. Louis, MO, USA). Mouse TGF-β, IL-10, IL-2, IFN-γ and IL-17A enzyme-linked immunosorbent assay (ELISA) kit was from ebiosciences. Recombinant mouse IL-17A was from R&D Systems. Anti-CD25-PE, anti-Foxp3-APC, anti-CD4-FITC and anti-IL-17-PE were all from BD Company (BD Bioscience, San Jose, CA, USA).

### Hepatoma H22-bearing mice and treatment

Male BALB/c mice (18–22 g), were purchased from Center of Medical Experimental Animals of Hubei Province (Wuhan, China). Animal experiments were conducted according to the Guide for the Care and Use of Laboratory Animals of Huazhong University of Science and Technology, as approved by the Animal Care Committee of Hubei Province, China (Approval Number: TY20120158). BALB/c mice were inoculated with H22 cells by subcutaneous injection of 2 × 10^6^ cells to the left flank. One day after implantation of tumor cells, the mice were divided into 4 groups. One group was administered with saline solution (0.9%) by intragastric administration each day (vehicle control group), and the other 3 groups (SBE group) was treated with SBE (50, 100, and 150 mg/kg/day) by intragastric administration for continuous 30 days. One day after implantation of tumor cells, the tumors were observed under 3D ultrasound every other day, and their volume was subsequently calculated by 3D ultrasound. To determine the recombinant mouse IL-17A effect on the tumor growth, IL-17A (0.5 μg per mouse) was injected into the peritoneal cavity of SBE treated mice for 2 weeks from day 9 after H22 inoculation.

### MTT assay for HepG2 cell proliferation

The proliferation of HepG2 cells after SBE treatment was assessed by MTT assay. Firstly, the SBE was dissolved in 50% DMSO to a stock concentration of 500 mg/ml and stored at −20 °C, and the working concentrations of SBE were made by diluting the stock solution in the cell culture medium. The final concentration of DMSO in the medium was < 0.5%. Secondly, cells were seeded into 96-well plates at the density of 3 × 10^3^ cells/well. After 12 h, the cells were treated with SBE in different concentrations (0.05, 0.1, 0.2, 0.3, and 0.5 mg/mL) for 48 h, respectively. Treatment with 0.5% DMSO was included as vehicle control. Thirdly, MTT were applied to each well after treatment. The supernatant were removed after 4 h incubation. Then, the DMSO were added to each. The supernatants were removed carefully and 150 μL of DMSO were added to each well. The formazan production was analyzed at 490 nm in a plate reader (Molecular Devices, LLC). The IC50 values were then calculated. This assay was performed in triplicate.

### NK cell cytotoxicity assay

The spleen from recipients was collected and pressed through nylon mesh to produce a single-cell suspension. NK cell (NK1.1^+^CD3^−^) were isolated with mouse NK cell isolation kit ((Miltenyi Biotec, Auburn, CA). Flow cytometric assay was used to assess the specific cytotoxicity of NK cells, and K562 cell was used as a target cell. Briefly, 2 × 10^6^ K562 (target cells) in 1 ml culture medium were incubated with 2 μl of CFSE (400 μM) for 10 min at 37 °C and washed with PBS three times. NK cells were mixed with 4 × 10^4^ labeled K562 (target cells) in a 24-well plate with indicated effector-to-target cell (E/T) ratios (10:1, 5:1). The mixture was centrifuged to enhance cell contact and incubated for 3 h. For the last 30 min of incubation, 10 μl of 200 μg/ml propidium iodide was added to the cells. Samples were analyzed by FCM (BD LSR-II) immediately. Each sample was prepared and analyzed in triplicate. The specific killing percentage was calculated by the following formula: [(%of target cell lysis-%of spontaneous death)/(100-% of spontaneous death)] × 100%.

### Isolation of tumor-infiltrating lymphocytes

Tumors were digested with collagenase and hyaluronidase for 1 h at 37 °C. After grinding with semifrosted slides and lysising of red blood cell, the dissociated cells were incubated on ice for 10 min, and then spun down at 1000 rpm for 2 min. The cell pellet was washed and used as tumor cells. The suspension cells were underlaid with 5 ml of lymphocyte-M solution, centrifuged at 2200 rpm for 30 min. Tumor-infiltrating lymphocytes were harvested from the interface for FCM staining.

After incubation of TILs at 4 °C with anti-mouse CD16/CD32mAb (2.4G2) in a staining buffer (phosphate-buffered saline containing 2% FCS and 0.1% sodium azide) on ice for 15 min. To detect regulatory T cells, cells were stained with anti-Foxp3-APC after surface staining with anti-CD25-PE and anti-CD4-FITC according to the the manufacturer’s protocols (BD Bioscience, San Jose, CA, USA).

To detect Th17 cells, TILs were stimulated for 4.5 h in the presence of 50 ng/ml PMA, 1 mg/ml ionomycin, and 2 mM monensin (all from Sigma-Aldrich) at 37 °C under 5% CO_2_. The cells were then stained for surface markers (anti-CD4-FITC) followed by labeling with anti-IL-17-PE cytokine Abs. Intracellular cytokine production was then analyzed by a LSR II flow cytometer (BD Bioscience, San Jose, CA, USA).

### Measurement of TGF-β, IL-10, IL-2, IFN-γ and IL-17A in the serum of mice by ELISA

The mice of control and SBE treatent groups were sacrificed, and the serum of mice were collected. The concentrations of TGF-β, IL-10, IL-2, IFN-γ and IL-17A in the serum were measured by ELISA using ELISA kits (Ebiosciences-Easy-Set-Go) according to the manufacturer’s protocols.

### Statistical analysis

Data are presented as mean ± SD. One-way analysis of variance was used for multiple comparisons, and Student’s *t*-test was used to compare two groups. *P*-values below 0.05 were considered as statistically significant.

## Results

### Identification of SBE by HPLC

**Fig. 1:**
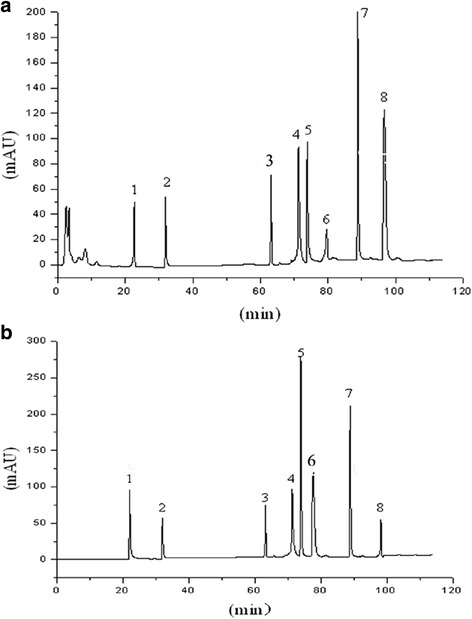
The HPLC chromatogram of SBE. **a** SBE; **b** mixed standard substances. 1: scutellarin; 2: naringin; 3: scutellarein; 4: luteolin; 5: apigenin; 6: wogonin; 7: scutebarbatine A; 8: scutebarbatine B

The components of SBE were identified by HPLC. As shown in Fig. 1, scutellarin, naringin, scutellarein, luteolin, apigenin, wogonin, scutebarbatine A, and scutebarbatine B were the main components of SBE and the retention times of these peaks were ranges from 20 min to 110 min.

### SBE treatment inhibited the proliferation of HepG2 cells

**Fig. 2:**
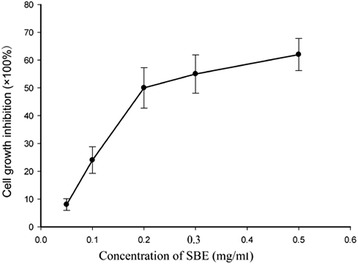
Inhibitory effect of SBE on the proliferation of HepG2 cells. Cells were treated with different concentrations of SBE. The cells viability were determined by the MTT assay. After 48 h treatment of SBE, we found that it exhibited a significant inhibitory effect on HepG2 cells with a dose-dependent manner, and there was significant difference among different dose of SBE (*P* < 0.05). Data are representative of three independent experiments

The proliferation of HepG2 cells was measured by MTT assay. After 48 h treatment of SBE, we found that it exhibited a significant inhibitory effect on HepG2 cells with a dose-dependent manner, there was significant difference among different dose of SBE (*P* < 0.05)., and the IC50 was about 0.20 ± 0.21 mg/ml (Fig. 2). DMSO treatment has no inhibitory effect on HepG2 cells.

### SBE treatment suppressed the tumor growth of hepatoma H22 tumor-bearing mice

**Fig. 3:**
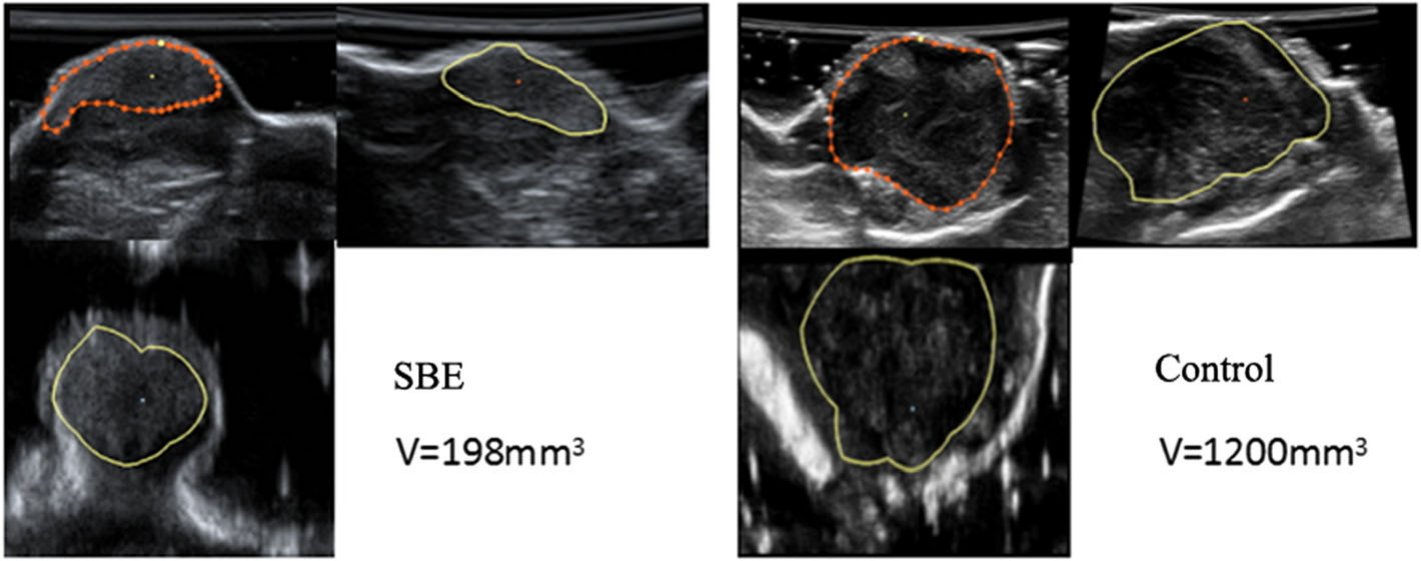
3D ultrasound pictures for tumor growth in SBE and saline solution treatment mice. Fifteen days after implantation of tumor cells, the tumors of SBE and control mice were observed under 3D ultrasound, and their volume was calculated by 3D ultrasound

**Fig. 4:**
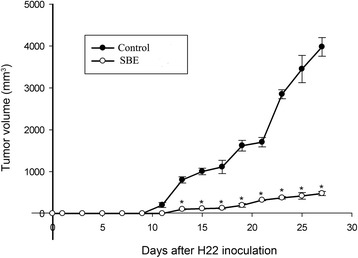
The effect of SBE treatment on the growth of H22 tumors. The volume of tumors of SBE group (50 mg/kg/day) was significantly smaller compared with the control group. Data are representative of three independent experiments. *P < 0.05 versus control

Hepatoma H22-bearing mice was used to verify the anticancer activities of SBE. The mice were administered with saline solution or SBE. One day after mice implantation of tumor cells, the tumors were observed under 3D ultrasound every other day, and their volume was subsequently calculated by 3D ultrasound (Fig. 3). As shown in Fig. 4, the volume of tumors of SBE group were significantly smaller compared with the control group (*P* < *0.05*).

### SBE treatment enhanced the NK cells’ cytotoxicity of hepatoma H22 tumor-bearing mice

**Fig. 5:**
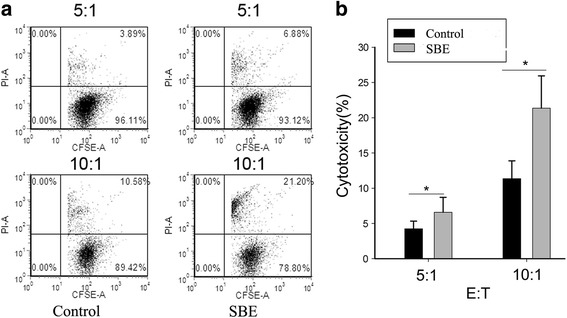
Cytotoxicity of NK cells is enhanced by SBE. **a** NK cells from control and SBE treated groups were incubated with indicated effector-to-target cell (E/T) ratios (5:1, 10:1). CFSE-positive cells were gated, and the percentage of PI-positive cells within this gate is shown. **b** Cytotoxicity of NK cells between the two groups with indicated effector-to-target cell (E/T) ratios (5:1, 10:1) is shown. This experiment was performed three times independently, yielding comparable results. *P < 0.05 versus control

The NK cell is important for anti-tumor and virus. Therefore, we characterized the cytotoxic effects of spleen NK cells on K562 cells. Spleen NK (DX5^+^CD3^−^) cells were isolated by microbeads and cytotoxicity assay was performed by FCM at different effector-to-target (E/T) ratios. As shown in Fig. 5, the cytotoxicity of NK cells against K562 cells was significant enhanced by SBE treatment (*P < 0.05*).

### SBE treatment reduced the amount of CD4 + CD25 + Foxp3+ regulatory T cells in tumor tissue

**Fig. 6:**
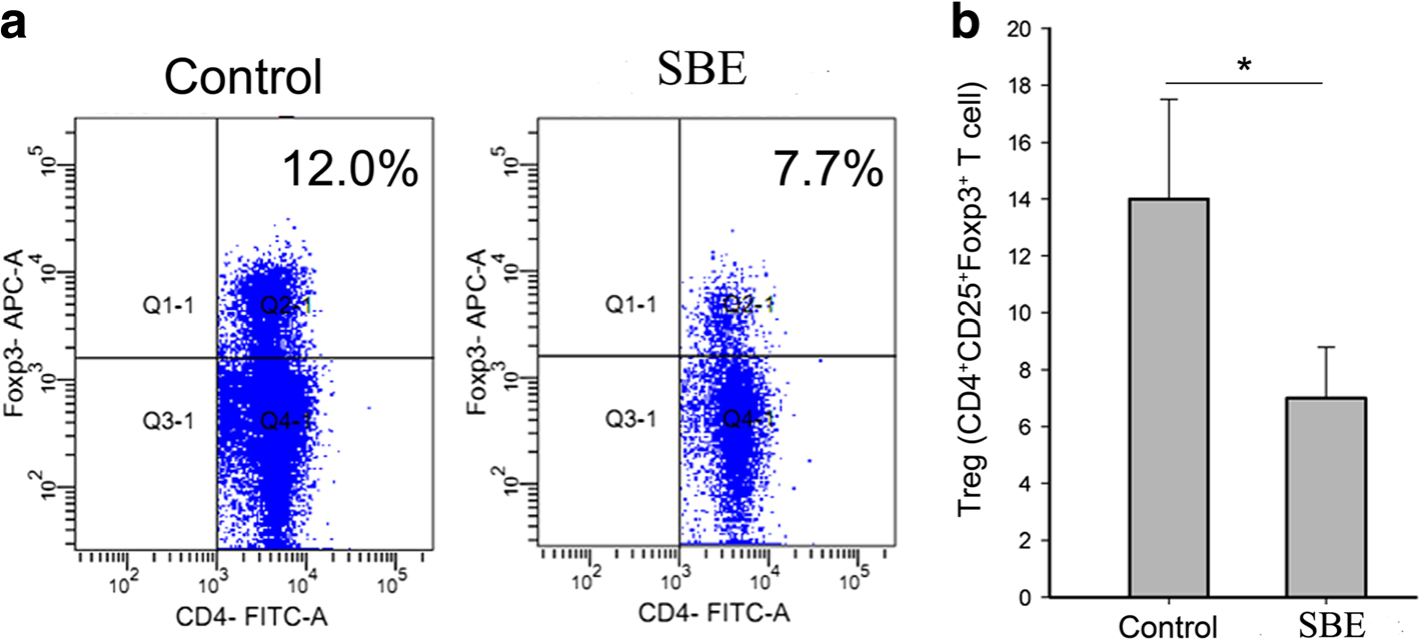
SBE treatment down-regulated the population of Treg cells. Tumors from control and SBE treated groups were digested with collagenase and hyaluronidase. **a** Tumor-infiltrating lymphocytes (TILs) were isolated, stained with anti-Foxp3-APC after surface staining with anti-CD25-PE and anti-CD4-FITC. **b** The amount of Treg cells in tumor microenvironment between the two groups is shown. Data are representative of three independent experiments. *P < 0.05 versus control

We investigated the effect of SBE on Treg cells infiltrating in tumor tissue. As shown in Fig. 6, the amount of Treg cells in tumor microenvironment was significantly decreased after SBE treatment (*P* < *0.05*). Therefore, these data suggested that SBE might regulate the infiltration of Treg cells in tumor microenvironment.

### SBE treatment reduced the amount of CD4^+^IL-17^+^ T cells (Th17 cell) in tumor tissue

**Fig. 7:**
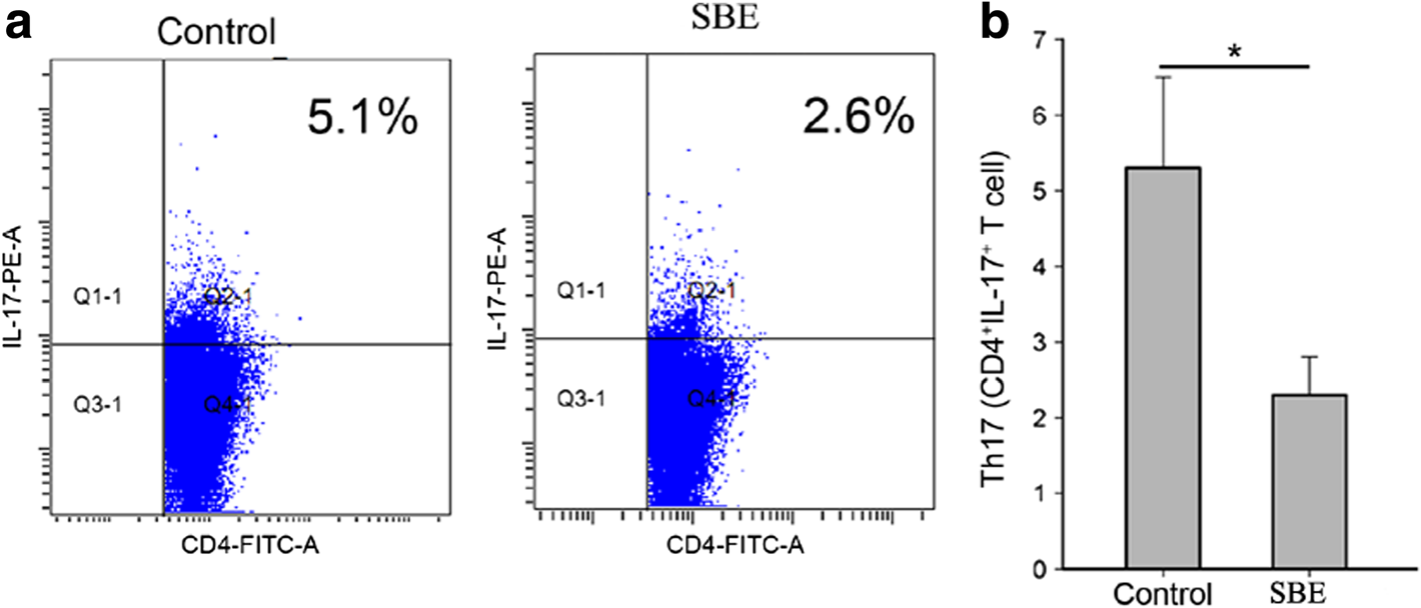
SBE treatment down-regulated the amount of CD4^+^IL-17^+^Th17 cells. **a** Tumor-infiltrating lymphocytes (TILs) were isolated and stimulated, and the cells were stained with anti-CD4-FITC followed by labeling with anti-IL-17-PE cytokine Abs. **b** The amount of Th17 cells in tumor microenvironment between the two groups is shown. Data are representative of three independent experiments. *P < 0.05 versus control

We investigated the effect of SBE on Th17 cells infiltrating in tumor tissue. As shown in Fig. 7, the amount of Th17 cells in tumor microenvironment was also significantly decreased after SBE treatment (*P* < 0.05). Therefore, these data suggested that SBE might regulate the infiltration of Th17 cells in tumor microenvironment.

### SBE treatment up-regulated Th1 cytokine and down-regulated Th17 and Treg related cytokine in serum of the hepatoma H22 tumor-bearing mice

**Fig. 8:**
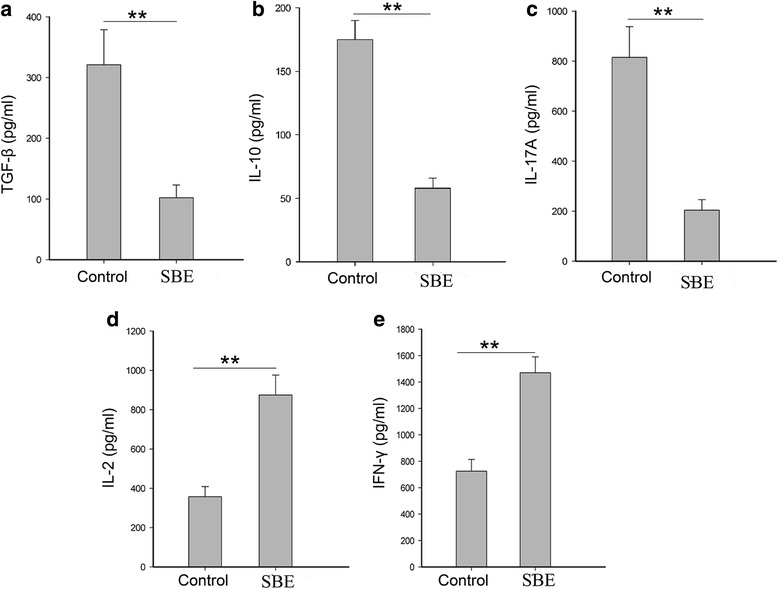
SBE treatment up-regulated Th1 cytokine and down-regulated Th17 and Treg related cytokine. The concentrations of TGF-β, IL-10, IL-17A, IL-2  and IFN-γ in the serum of mice were measured by ELISA. The amount of TGF-β (**a**), IL-10 (**b**), IL-17A (**c**), IL-2 (**d**) and IFN-γ (**e**) between the two groups is shown respectively. Data are representative of three independent experiments. **P < 0.01 versus control

The concentrations of TGF-β, IL-10, IL-2, IFN-γ, and IL-17A in the serum of control and SBE treated mice were measured by ELISA. As shown in Fig. 8, SBE treatment significantly down-regulated Th17 and Treg related cytokine, IL-17 (Fig. 8c), TGF-β (Fig. 8a), IL-10 (Fig. 8b) in the serum of tumor bearing mice (*P* < 0.01). On the contrary, Th1 related cytokine (IL-2, IFN-γ) was significantly up-regulated in the serum of tumor bearing mice (*P* < 0.01).

### Recombinant IL-17A administration reversed the anti-tumor effect of SBE

**Fig. 9:**
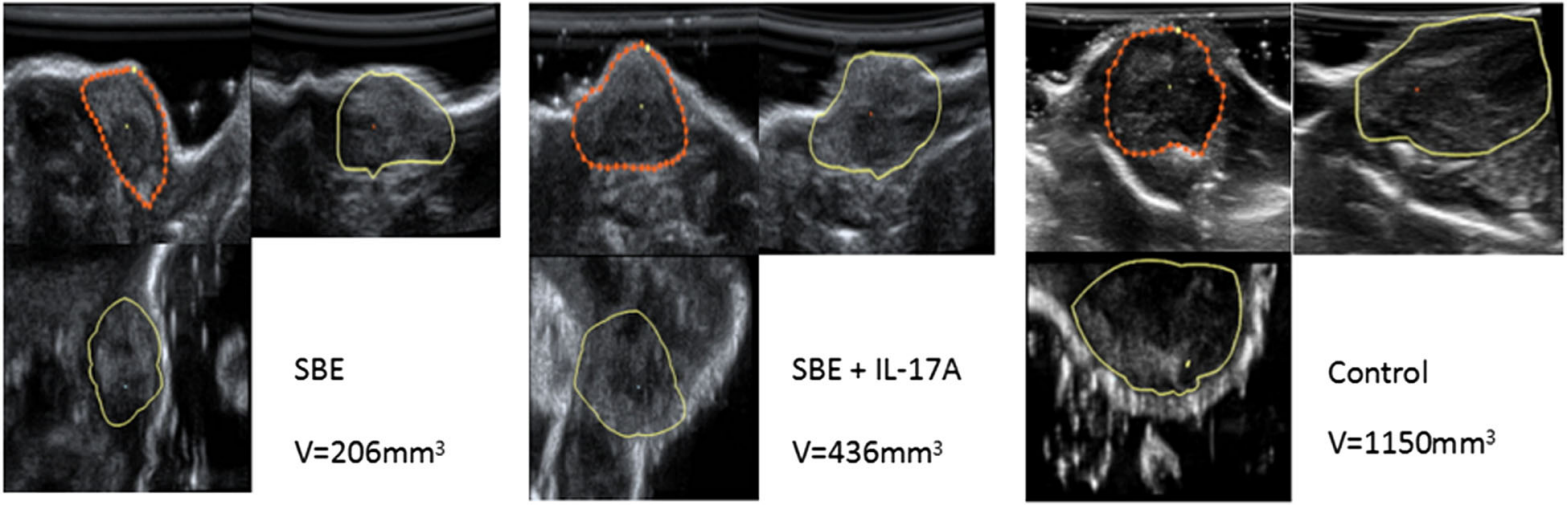
3D ultrasound pictures for tumor growth in SBE, saline solution, and SBE combined with IL-17A treatment mice. Fifteen days after implantation of tumor cells, the tumors of SBE, control, and IL-17A combined with SBE treatment mice were observed under 3D ultrasound, and their volume was calculated by 3D ultrasound

**Fig. 10:**
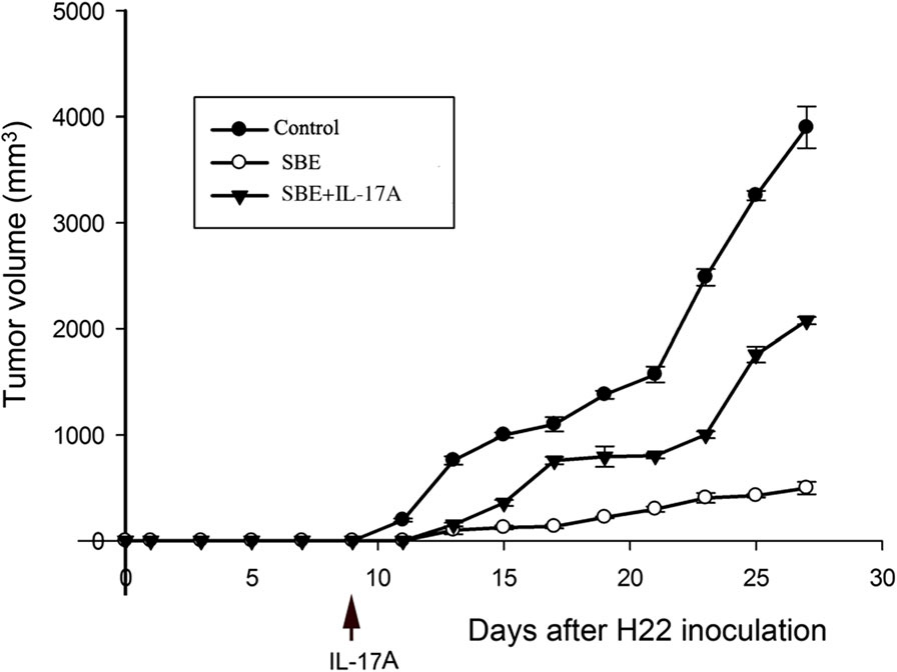
Recombinant IL-17A administration reversed the anti-tumor effect of SBE. Tumors volume of SBE combined with IL-17A treatment group was significantly larger than SBE treatment group (*P* < 0.05), and significantly smaller than control group (*P* < 0.05)

One day after mice implantation of tumor cells, the tumors were observed under 3D ultrasound every other day, and their volume was subsequently calculated by 3D ultrasound (Fig. 9). As shown in Fig. 10, recombinant IL-17A administration could reverse the anti-tumor effect of SBE, and the volume of tumors of SBE combined with IL-17A treatment group was significantly larger than SBE treatment group (*P* < 0.05), and significantly smaller than vehicle control group (*P* < 0.05).

## Discussion

The immune system plays an important role in anti-tumor defense. Progressive hepatocellular tumor growth is frequently accompanied by a concomitant immunosuppression regardless of tumor location and etiology [21]. Tumors have evolved numerous immune escape mechanisms: down-regulating of surface MHC class I molecules to escape NK cells’ killing; the generation of cells with suppression functions, including regulatory cells (Treg) and myeloid-derived suppressor cells [22]. Therefore, based on its pathogenesis as well as a number of correlative studies, immunotherapy represents a potential therapeutic option for patients with HCC [23].

Many reports suggested that the antitumor activity by several traditional Chinese herbs as mediated via augmentation of the immune response [24–26]. Therefore, we investigated the effect of traditional medicine, SB on immunity. Hepatoma H22-bearing mice were used to elucidate the immunomodulatory function of anti-tumor activity.

The human HCC cell line, HepG2 cells has been extensively used for vitro experiment [27, 28]. Based on the previous studies [29–31], we selected different concentrations of SBE in our study, and examined their effects on HepG2 cell in vitro and the tumor growth in hepatoma H22-bearing mice. The results of this study showed that SBE could not only inhibit the proliferation of HepG2 in vitro, but also inhibit the tumor growth in hepatoma H22-bearing mice. It demonstrates that SBE has the inhibitory effects on the tumor growth.

In this study, we investigated the underlying immunomodulatory function of SBE on the tumor growth of hepatoma H22-bearing mice. Natural killer cells (NK cell) belong to the innate immune system and play a critical role in the host defense against cancer [32]. NK cells represent one major component of the liver microenvironment. In addition to direct killing of tumor cells, NK cells are able to rapidly release immunomodulatory cytokines, which activate leukocytes of both the innate and adaptive immune system. Unlike CTLs, however, the killing by NK cells is non-specific and NK cells do not need to recognize antigen/MHC on the target cell. NK cells can react against and destroy target cell without prior sensitization to it. The results of our study showed SBE treatment significantly enhanced the killing activity of NK cells from splenocytes in H22 tumor-bearing mice. It suggested that SBE treatment could enhance NK cells’ killing tumor ability.

Regulatory T cells characterized by the expression of the transcription factor Foxp3 play a pivotal role in immune homeostasis and suppress function of effector cells such as CD4^+^ T cells, CD8^+^ T cells, and natural killer (NK) T cells [33]. Previous study [34] has demonstrated that an abundant accumulation of Treg cells was found in tumor regions compared with nontumor regions in HCC patients. Our study also found that the amount of CD4^+^CD25^+^Foxp3^+^ regulatory T cells in tumor tissue was significantly decreased in SBE treated group. This result confirmed the view that tumor cells can recruit these Treg cells to inhibit the efficiency of cancer immunotherapy.

The production of IL-17 characterizes a subset of CD4+ helper T cells (Th17 cells). T helper 17 (Th17) cells are an important inflammatory component and have been shown to promote inflammation in a number of autoimmune diseases [35]. The development of Th17 cells is distinct from the development of Th1, Th2 and regulatory T cells and is characterized by unique transcription factors and cytokine requirements. A previous study [20] reported that Th17 cells were significantly increased in tumors of HCC compared with corresponding non-tumor regions, accumulation of intratumoral IL-17-producing cells may promote tumor progression through fostering angiogenesis, and intratumoral IL-17-producing cell could serve as a potential prognostic marker and a novel therapeutic target for HCC. The results of our study showed that SBE treatment significantly decreased the amount of CD4^+^IL-17^+^ (Th17) cells in the tumor tissue. This may partially cause the inhibition of tumor growth.

NK cell, Treg and Th17 cells exert their function through cytokine secreted in the tumor microenvironment. Therefore, we detected the Th1, Treg and Th17 related cytokine in the serum of H22 tumor-bearing mice. The results of our study showed SBE treatment up-regulated Th1 cytokine (IL-2 and IL-12p70) and down-regulated Th17 (IL-17) and Treg (TGF-β and IL-10) related cytokine in the serum of tumor bearing mice. The main producing cytokine of Th17, recombinant IL-17A administration could reverse the anti-tumor effects of SBE. It suggested that Th17 could partially cause the tumor growth, and SBE might inhibit the tumor growth through the intervention of Th17 cell.

The present study has limitations. First, we tested only three dosages of SBE in inhibiting the growth of hepatoma H22 cells in vivo. More experiments are needed to explore other dosages of SBE, and there might be a more appropriate dosage of SBE which is also effective in inhibiting the growth of hepatoma H22 cells in vivo. Second, SBE using in our study included many components. More experiments are needed to explore which component is the most important for inhibition of tumor growth.

## Conclusion

In summary, SBE could inhibit the proliferation of HepG2 cells in vitro. Furthermore, SBE also could inhibit the growth of H22 implanted tumor in hepatoma H22-bearing mice, and this function might be associated with immunomodulatory activity through down-regulating of Treg cells and manipulating Th1/Th17 immune response.

## Abbreviations

ELISA: Enzyme-linked immunosorbent assay
HCC: Hepatocellular carcinoma
HPLC: High performance liquid chromatography
MTT: 3-(4,5-dimethylthiazol-2-yl)-2,5-diphenyltetrazolium bromide
SB: *Scutellaria barbata*
